# RNA Through Time: From the Origin of Life to Therapeutic Frontiers in Transcriptomics and Epitranscriptional Medicine

**DOI:** 10.3390/ijms26114964

**Published:** 2025-05-22

**Authors:** Cecilia Martínez-Campos, Humberto Lanz-Mendoza, Jorge A. Cime-Castillo, Óscar Peralta-Zaragoza, Vicente Madrid-Marina

**Affiliations:** 1 National Institute of Genomic Medicine (Instituto Nacional de Medicina Genómica, INMEGEN), Periférico Sur 4809, Ciudad de México 14610, Mexico; ccampos@inmegen.edu.mx; 2 Center for Research on Infectious Diseases, National Institute of Public Health (Instituto Nacional de Salud Pública, INSP), Universidad 655, Cuernavaca 62100, Mexico; humberto@insp.mx (H.L.-M.); jorge.cime@insp.mx (J.A.C.-C.); operalta@insp.mx (Ó.P.-Z.)

**Keywords:** RNA, therapeutic vaccines, miRNAs, siRNAs, RNA modifications

## Abstract

This review examines the evolutionary trajectory and functional versatility of RNA, beginning with its proposed involvement in the origin of life and culminating in its current application in therapeutic strategies. We explored the complexity of the transcriptome, splicing mechanisms, and the regulatory functions of non-coding RNAs, especially microRNAs. The processes underlying microRNA biogenesis and activity are discussed in the context of their potential as therapeutic tools. Advances in RNA-based technologies have been further illustrated by the development of mRNA vaccines, representing a significant breakthrough in biomedical innovation. Additionally, we explored the growing field of epitranscriptomics—chemical modifications to RNA that modulate its stability, translation, and function—by analyzing the roles of modification enzymes known as writers, erasers, and readers. Focus is given to how these alterations influence immune reactions and guide the strategic development of future modified mRNA vaccines. Collectively, these advances underscore RNA’s multifaceted roles and its transformative potential in the biological and medical sciences.

## 1. RNA in the Origin of Life

The origin of life continues to be one of the most fundamental questions in biology with various theories proposed to explain this phenomenon. Among these, the RNA world hypothesis stands out, suggesting that RNA was the initial molecule responsible for storing and transmitting genetic information, playing a crucial role in life’s beginnings on Earth. This theory proposes that RNA’s unique dual role, as both a carrier of genetic information and a catalytic molecule, enabled it to self-replicate and evolve, ultimately leading to the development of DNA and proteins. Experimental evidence suggests that RNA can spontaneously assemble under conditions resembling those of the early Earth, and its fundamental components, nucleotides, have been identified in meteorites and created in laboratory experiments simulating primordial environments [1,2]. The discovery of ribozymes and RNA molecules with enzymatic properties further supports the RNA world hypothesis [3]. Furthermore, it has been demonstrated that RNA can catalyze replication, ligate RNA strands, and even synthesize peptides [4,5].

The RNA world hypothesis posits that the multifunctional capabilities of RNA enabled it to assume a pivotal role in the origin of life. Unlike DNA, RNA possesses some characteristics that make it a plausible candidate for the earliest self-replicating systems, including the following:

1. Replication and catalysis: RNA possesses the ability to self-replicate and catalyze chemical reactions, whereas DNA necessitates the presence of proteins for replication and catalysis [6].
2. Structural flexibility: The single-stranded nature of RNA allows it to form complex three-dimensional structures, facilitating its ability to perform various functions including catalysis [7].
3. Spontaneous synthesis: RNA nucleotides can be synthesized spontaneously in primordial environments, whereas DNA synthesis requires complex chemical pathways [2].
4. Versatility: RNA functions as a genetic material, catalyst, and structural component, whereas DNA primarily serves as a repository for genetic information [8].
5. Error tolerance: A higher mutation rate of RNA facilitates rapid evolution and adaptation, which is advantageous in primordial environments [4].

The finding that enzymes are not exclusively proteins was established by Thomas Cech and Sidney Altman in the 1980s [9,10]. This discovery, which highlights the catalytic capabilities of ribonucleic acids, represents a significant advancement with profound implications for the understanding of prebiotic synthesis [11]. One remarkable feature of RNA is that specific RNA molecules, known as ribozymes, exhibit catalytic properties similar to those of proteins. The discovery of ribozymes demonstrated that RNA is not solely a template for protein synthesis but can also catalyze biochemical reactions [12,13]. This finding offers a compelling explanation for how life might have originated in the absence of enzymes [14]. It was subsequently proposed that the initial phase of evolution progressed from RNA molecules capable of performing catalytic functions required for assembly from a soup of nucleotides [14].

The environmental conditions of the early Earth were drastically different from those today. It is believed that the primordial soup—rich in organic compounds—provided a suitable environment for the formation of RNA. Laboratory experiments such as the Urey experiment [15] and other simulations [16] have shown that amino acids and nucleotide precursors can form under conditions similar to those on early Earth. Additionally, research has suggested that RNA could have emerged from simple building blocks on primordial Earth when the conditions were appropriate. RNA molecules have been shown to act both as templates and catalysts in their own replication, fulfilling the operational definition of a self-duplicating entity [17]. The catalytic capabilities of RNA are now known to be remarkably diverse [18], and riboswitches, regulatory RNA elements, have been identified across a wide range of organisms, including archaea, bacteria, plants, fungi, and algae [19]. A self-replicating system is characterized by a molecule’s ability to direct the covalent assembly of its components to produce identical copies. When the product can also catalyze the same process, the system becomes autocatalytic. A notable example involves a redesigned R3C ligase ribozyme engineered to catalyze the ligation of two RNA substrates into a copy, demonstrating an RNA-catalyzed RNA ligation reaction [20]. This replication process is entirely dependent on the catalytic activity of the RNA, illustrating a protein-independent mechanism for molecular self-replication with implications for understanding early genetic systems (Table 1).

Although the RNA world hypothesis is generally accepted, it faces specific criticisms and obstacles. Some researchers contend that the instability of RNA and its inefficiency in early environmental conditions make it improbable that it played a crucial role in the origin of life. Advances in structural biology and comparative genomics have shed light on this phenomenon. Current evidence suggests that evolution proceeded in distinct stages, beginning with an RNA world, progressing to a mixed RNA/protein world, and ultimately culminating in a DNA-based world [21]. The evolution of the RNA world has been suggested to occur within the context of competing cells and viruses, with viruses playing a crucial role in the shift from RNA to DNA [21]. Currently, researchers are exploring new pathways for prebiotic chemical synthesis of ribonucleotides, and additional experiments are being conducted to isolate ribozymes from random RNA sequences [22]. As research continues, understanding how RNA may have contributed to the origin of life can provide profound insights into the beginnings of all biological systems and the nature of life itself.

However, despite its compelling features, the RNA world hypothesis faces several challenges:

1. Stability and Complexity: RNA is relatively unstable compared to DNA, and the complexity of forming functional ribozymes in a prebiotic environment is a significant challenge.
2. Transition to the DNA/Protein World: The transition from an RNA-based world to one utilizing DNA and proteins raises questions about how such a shift occurred and what mechanisms facilitated it.
3. Alternative Theories: Other hypotheses, such as the role of peptide nucleic acids (PNAs) or the involvement of other polymers, also explore possible pathways for the origin of life.

The RNA world hypothesis presents a compelling framework for understanding the origin of life, proposing that early life forms may have relied on RNA as both a genetic material and a catalyst for biochemical processes. Moreover, the evolutionary relationships among modern organisms, where RNA plays a central role in cellular functions, further reinforce the idea that RNA could have been crucial in the transition from simple molecules to complex life. Although the RNA world hypothesis does not account for every aspect of life’s origin, it provides a coherent and testable model that continues to shape our understanding of the molecular evolution and origin of life on Earth.

## 2. Technical Improvements in RNA Extraction

One of the most significant advancements in experimental biology has been the improvement in RNA extraction techniques. In the early days of molecular biology, studying gene expression was challenging due to the extreme instability and rapid degradation of RNA. The introduction of RNase inhibitors was pivotal in overcoming this hurdle and advancing RNA extraction methods. The RNA extraction technique using TRIzol, which is based on guanidinium isothiocyanate, a chaotropic agent, was developed following the method described by Chomczynsky and Sacchi in 1987 [23]. This breakthrough has enabled more efficient and reliable RNA isolation, significantly advancing gene expression and molecular biology research.

Over the past 30 years, the field of RNA biology has experienced significant growth, with the publication of hundreds of thousands of research papers, facilitated by advancements in total RNA experimental techniques. For instance, a recent study compared four methodologies for extracting SARS-CoV-2 RNA from wastewater. These methods include cetyltrimethylammonium bromide (CTAB)-based, TRIzol-based, and guanidinium thiocyanate (GTC)-based extraction procedures coupled with silica spin-column-based purification. After RNA extraction, virus recovery rates were compared using RT-qPCR-based detection. The results showed that the CTAB-PVP-column and TRIzol-column methods had the highest recovery rates, yielding significantly higher recovery rates than both GTC-based and automated extraction methods [24]. These findings offer valuable information on the performance of commonly used viral RNA extraction/purification procedures, and thus guide method selection for efficient virus detection in complex sewage matrices. Efficient extraction and purification of RNA to ensure optimal RNA quantity and quality is critical for successful RNA detection [24]. For example, these technical advancements have facilitated physiological studies, allowing researchers to identify transduction signals involved in the differentiation of CD4/CD8 double-positive lymphocytes into single CD4 or CD8 cells during intrathymic development, a process critical for immune response [25]. Furthermore, technical advancements in RNA research over the years have revolutionized our ability to manipulate and study RNA in the laboratory and deepened our understanding of its potential role in the origin of life.

## 3. Transcriptome

RNA is a single strand of nucleic acid composed of a chain of ribonucleotides (nucleosides linked to 5′-ribose). The ribonucleotides in RNA include adenine, guanine (both purine bases), cytidine, and uracil (both pyrimidine bases). The transcriptome “refers to the whole set of RNAs transcribed (coding RNA 1–2% and No-coding RNAs 98%) by the genome from a specific tissue or cell type at a particular developmental stage or under certain physiological circumstances”. Transcriptomic analysis involves sequencing of all RNA transcripts in whole blood, tissues, or individual cells. Following RNA sequencing, transcriptome analysis enables us to understand genome expression at the RNA level, offering insights into gene expression regulation, gene products, and physiological dynamics [26].

The transcriptome, the complete repertoire of transcripts in a species, represents a clear link between the information encoded by DNA and phenotype. Based on their functions, RNA can be classified as follows:

(A) Protein synthesis

Messenger RNA (mRNA): Single-stranded RNA molecules act as intermediates between DNA and the process of protein synthesis. They transport genetic information transcribed from DNA in the nucleus to ribosomes located in the cytoplasm, where they function as templates for protein translation [27,28,29]. During transcription, RNA polymerase synthesizes a complementary mRNA strand from a DNA template. In eukaryotic cells, the resulting primary transcript, known as pre-mRNA, undergoes several processing steps such as 5′ capping, splicing, and 3′ polyadenylation to become mature mRNA [30,31,32,33,34]. In the translation stage, the mature mRNA is transported to the cytoplasm and translated by ribosomes [35]. Each three-nucleotide codon on the mRNA specifies a particular amino acid, directing the assembly of polypeptides with the help of transfer RNA and ribosomal RNA [36]. Consequently, mRNA is crucial in gene expression, as it transmits genetic information from DNA to the cellular machinery that produces proteins [37].

Transfer RNA (tRNA): These are adaptor molecules, typically composed of 76 to 90 nucleotides (nt), characterized by a conserved cloverleaf-like secondary structure. They play a role in translating the genetic code into functional proteins by delivering specific amino acids to the ribosome in accordance with the codon sequence of the mRNA [38]. tRNAs have a conserved structure with key functional domains: the acceptor stem, ending in a CCA sequence, serves as the aminoacylation site for amino acid attachment; the anticodon arm enables codon recognition on mRNA; the D-arm and TψC arm support proper folding, enzyme recognition, and ribosome interaction; and the variable loop contributes to tRNA identity [39].

Beyond their canonical role in translation, tRNAs can also be processed into evolutionarily conserved small RNA fragments, known as tRNA-derived fragments (tRFs). These molecules are generated through precise cleavage of either mature tRNAs or their precursor transcripts and have been identified across a wide range of organisms [40]. These fragments are produced in both cytoplasmic and mitochondrial compartments and are classified into at least four structural types: 5′-tRFs, 3′-tRFs, internal tRFs (i-tRFs), and tRNA halves (tiRNAs) [41]. Emerging evidence suggests that tRFs participate in various regulatory processes, including gene silencing, epigenetic inheritance, stress responses, modulation of immune functions, and tumor progression [42,43].

Ribosomal RNAs (rRNA): These are essential non-coding RNA molecules that form the core structural and catalytic components of ribosomes, the molecular machines responsible for protein synthesis in all living cells. rRNAs perform critical functions by stabilizing ribosomal structure and catalyzing key biochemical reactions. Most notably, rRNA acts as a ribozyme, catalyzing the formation of peptide bonds during the elongation phase of translation [44,45]. Each ribosome contains three functional binding sites for tRNA, which coordinate the stepwise addition of amino acids to the growing polypeptide chain. The A site (aminoacyl site) is where incoming aminoacyl-tRNAs, charged with specific amino acids, bind. The P site (peptidyl site) holds the tRNA carrying the nascent polypeptide chain. Once the amino acid has been transferred, the uncharged tRNA moves to the E site (exit site) and is released from the ribosome [46,47].

In eukaryotic cells, the 45S precursor rRNA (pre-rRNA) is transcribed by RNA polymerase I within the nucleolus and subsequently processed into three mature rRNA species: 18S, 5.8S, and 28S rRNAs. These rRNAs constitute the essential structural and functional components of ribosomes. In contrast, the 5S rRNA is transcribed by RNA polymerase III (Pol III) outside the nucleolus. Together, these four rRNAs, 18S, 5.8S, 28S, and 5S, assemble with ribosomal proteins to form two structural components of the eukaryotic ribosome: the 40S small subunit, which includes the 18S rRNA and approximately 33 ribosomal proteins, and the 60S large subunit, which incorporates the 5S, 5.8S, and 28S along with about 47 ribosomal proteins. These subunits combine to form a functional 80S ribosome, which is responsible for protein synthesis in eukaryotic cells [47,48].

(B) Non-coding regulatory RNA

MicroRNAs (miRNAs): These are a class of small, single-stranded, non-coding RNAs, typically 19–25 nucleotides long that form hairpin structures. They are evolutionarily conserved across a wide range of eukaryotic species and have also been identified in various viruses. miRNAs play crucial roles in post-transcriptional regulation of gene expression by binding to target messenger RNAs (mRNAs), leading to translational repression or mRNA degradation [49]. Approximately 50% of miRNA genes are located in genomic regions susceptible to structural changes, such as fragile sites, regions of loss of heterozygosity, or amplification. This localization suggests a potential link between miRNA gene positioning and genomic instability, which may have implications in various diseases, including cancer [50].

miRNAs exert their regulatory functions by incorporating into the RNA-induced silencing complex (RISC) and guiding it to target mRNAs. The binding typically occurs at the 3′ untranslated regions (3′-UTRs) of target mRNAs through a “seed sequence” located at positions 2–8 from the 5′ end of the miRNA [51]. RISC mediates the cleavage and subsequent degradation of mRNA when perfect complementarity exists between a miRNA and its target, RISC mediates the cleavage and subsequent degradation of the mRNA. In the case of partial complementarity, miRNAs inhibit gene expression by repressing translation without degrading the mRNA [52].

Short interfering RNAs (siRNA): These are a class of double-stranded RNA molecules, typically 20–30 nucleotides long, that play pivotal roles in the RNA interference (RNAi) pathway [53]. SiRNAs can be broadly classified into two types: endogenous (endo-siRNAs) and exogenous (exo-siRNAs). Endo-siRNAs are generated from internal genomic sources, such as transposons or repetitive sequences, and contribute to gene regulation and protect against genomic instability. In contrast, exo-siRNAs originate from external sources such as viral infections or synthetic double-stranded RNA molecules [54]. Upon entering the cell, siRNAs are incorporated into RISC, binding and cleaving target mRNAs with perfect sequence complementarity, and preventing their translation into proteins [55]. siRNAs can be chemically synthesized and introduced into cells via transfection, making them powerful tools for experimental gene knockdown, providing a therapeutic potential in treating conditions such as chronic diseases, viral infections, cancer, among other conditions [55,56,57].

Piwi-interacting RNAs (piRNAs): These constitute the largest class of small non-coding RNA molecules and exert their function by forming RNA–protein complexes with P-element-induced wimpy testis (PIWI) proteins, a subfamily of Argonaute proteins. piRNAs were first discovered in *Drosophila melanogaster* [58], and have since been identified in a wide range of species, including *Caenorhabditis elegans*, zebrafish, mice, and humans [59]. These piRNA–PIWI complexes primarily mediate the epigenetic and post-transcriptional silencing of transposable elements and other repetitive or aberrant transcripts, particularly in germline cells [60,61,62]. piRNAs also contribute to the regulation of gene expression and the maintenance of genomic integrity in the germline, underscoring their broader significance in safeguarding genome stability [63,64].

piRNAs are predominantly generated from specialized genomic regions known as piRNA clusters, which act as transposon traps, enabling the recognition and silencing of transposable elements through an RNA-based adaptive immune-like mechanism [65]. Compared to miRNAs, piRNAs are generally longer (24–32 nucleotides), display greater sequence diversity, and are produced following a biogenesis pathway that involves Zucchini-dependent cleavage and amplification via the ping-pong cycle [66,67].

Circular RNAs (circRNA): These are a class of single-stranded RNA molecules distinguished by their covalently closed-loop structure, in which the conventional 5′ and 3′ ends of linear RNAs are joined together [68]. This unique topology confers several important properties, most notably increased stability, as the absence of free ends renders circRNAs resistant to exonuclease-mediated degradation.

circRNAs can be classified into several types, including exonic circRNAs (EcRNAs), exon–intron circRNA (ElciRNAs), and circular intronic RNAs (CiRNAs) [69]. Initially, circRNAs were categorized as non-coding RNAs due to their lack of a 5′ cap and 3′ poly(A) tail, a feature typically required for translation. However, recent evidence indicates that some circRNAs do possess protein-coding potential [69]. They can function as microRNA or protein inhibitors (sponges), scaffold molecules, or transcription modulators [70]. Due to their abnormal expression in various human tumors, circRNAs have attracted significant interest as potential targets for the development of RNA-based therapeutic strategies [71].

(C) Small nucleolar RNA (snoRNA): These are small non-coding RNAs that are widely present in the nucleoli of eukaryotic cells [72]. SnoRNAs are mainly encoded by the intronic regions of both protein-coding and non-protein-coding genes [73,74,75,76]. SnoRNAs are generally classified into three main groups based on their structure and function. C/D box snoRNAs (SNORDs) are typically 60–90 nucleotides long and contain conserved C (RUGAUGA) and D (CUGA) motifs; H/ACA box snoRNAs (SNORAs) are usually 120–140 nucleotides in length and are defined by the presence of an H box (ANANNA) and a terminal ACA motif, and a third group, known as small Cajal body-specific RNAs (scaRNAs or SCARNAs), containing both C/D and H/ACA box motifs in diverse combinations [77,78,79].

SnoRNAs direct chemical modifications on rRNA. In mammals, SNORDs guide site-specific 2′-O-methylation, catalyzed by the methyltransferase Fibrillarin [80,81,82], in association with a snoRNP complex that includes the proteins NOP56, NOP58, and 15.5K [83,84]. In contrast, SNORAs guide site-specific pseudouridylation, catalyzed by the pseudouridine synthase Dyskerin [85,86], together with a snoRNP complex containing NHP2, NOP10, and GAR1 [87,88,89]. These modifications are critical for the proper folding, structural stability, and functional activity of rRNAs [90].

(D) Small nuclear RNAs (snRNA): These are a type of short RNA molecules found in the splicing speckles and Cajal bodies inside the nuclei of eukaryotic cells, which are synthesized by either RNA polymerase II (Pol II) or III [91,92,93,94,95]. Their primary role is to process heterogeneous nuclear RNA (hnRNA). Small nuclear RNAs (snRNAs) collaborate with specific proteins to form small nuclear ribonucleoproteins (snRNPs), which are essential components of the spliceosome. The core of the spliceosome is U-rich, U1 (164 nt), U2 (191nt), U4 (141nt), U5 (116 nt), and U6 (107 nt) snRNAs, each having a unique function in identifying and excising introns during the splicing process [96,97,98,99,100,101].
(E) Long non-coding RNAs (long ncRNAs, lncRNA): These are a class of RNA transcripts longer than 200 nucleotides that exhibit little or no protein-coding potential [102]. These molecules can regulate gene expression through diverse mechanisms. Some lncRNAs act in cis, modulating the activity of nearby genes by recruiting chromatin-modifying complexes to specific genomic loci, thereby promoting either transcriptional activation or repression [103,104]. Others function in trans, influencing the expression of distant genes. A well-characterized example is HOTAIR (HOX transcript antisense RNA), which is transcribed from the HOXC locus but represses transcription at the HOXD locus through epigenetic silencing mechanisms [105]. Additionally, lncRNAs play structural roles in the nucleus, contributing to the organization of nuclear architecture and the formation of subnuclear domains such as paraspeckles and nuclear speckles, ultimately influencing gene expression [106,107]. Over 100,000 human lncRNAs have been identified [108,109,110]; however, the functions and biological significance of most of them remain largely uncharacterized.

**Figure 1:**
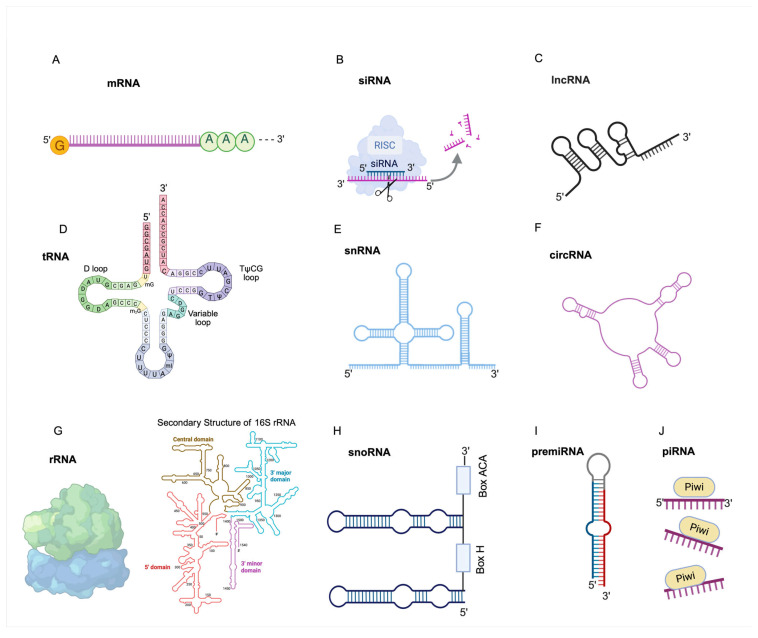
Major coding and non-coding RNA types. (**A**) Messenger RNA (mRNA). At the 5′ end, the mRNA is modified with a 7-methylguanosine cap (m^7^G cap), which facilitates ribosome binding and protects the transcript from exonuclease degradation. At the 3′ end, the mRNA features a polyadenylated tail (poly(A) tail) consisting of approximately 100–250 adenosine residues. (**B**) Small interfering RNAs (siRNA) are RNA molecules involved in RNA interference, guiding sequence-specific degradation of complementary mRNAs to regulate gene expression. (**C**) Long non-coding RNAs (lncRNA) are non-protein-coding transcripts longer than 200 nucleotides that regulate gene expression. (**D**) Transfer RNA (tRNA). In addition to the standard bases—adenine (A), uracil (U), guanine (G), and cytosine (C)tRNAs incorporate a variety of modified nucleotides formed post-transcriptionally. Examples include D (dihydrouridine), mI (methylinosine), mG (methylguanosine), m^2^G (dimethylguanosine), and Ψ (pseudouridine), which contribute to tRNA stability and function. (**E**) Small nuclear RNAs (snRNA) are core components of the spliceosome complex. They facilitate the precise removal of introns and the joining of exons, enabling the production of mature mRNA transcripts. (**F**) Circular RNA (circRNA) are covalently closed-loop RNA molecules that may function as regulators of transcription. (**G**) Ribosomal RNAs (rRNA) are structural and catalytic components of ribosomes essential for protein translation. (**H**) Small nucleolar RNAs (snoRNA). A representative schematic of an H/ACA box snoRNA is shown, illustrating the characteristic hairpin–hinge–hairpin–tail structure with conserved H (ANANNA) and ACA motifs. (**I**) premiRNA. The 5′ and 3′ ends of the pre-miRNA are shown, along with the terminal loop and base-paired stem region. This structure is recognized and processed in the cytoplasm by Dicer into a ~22-nucleotide miRNA duplex. (**J**) piRNA. piRNAs are single-stranded piRNA molecules, typically 24–31 nucleotides in length, associated with a PIWI protein.

A general representation of RNA molecules is shown in Figure 1.

RNA has a high propensity to form intramolecular helices and tertiary structures, which are central to the functionality of a given RNA molecule. Over the last several decades, major advances have been made in the understanding of RNA folding and structure. Proper folding is essential for small ribozymes to adopt catalytically active RNA structures. Crystallographic data, structural and thermodynamic analyses, chemical and enzymatic footprinting, thermal denaturation, and mutagenesis, among other techniques, have shown that the formation of correct secondary and tertiary interactions, divalent ion and solvent interactions, and proper folding of the catalytic core and substrate-binding region are fundamental for ribozyme activity [111,112,113,114,115].

mRNA is synthesized in the nucleus and associates during its synthesis with RNA-binding proteins (RBPs), which are ubiquitous partners of cellular RNA, where 1 to 2% are coding mRNA and 98% are non-coding RNA [116]. Together, they form dynamic ribonucleoprotein (RNP) particles, often in highly combinatorial fusion. RBPs regulate the metabolism of RNA within a cell and control all aspects of RNA fate. Defects in their functions underlie a broad spectrum of human pathologies. RBPs are critically important for RNA function in terms of structural, regulatory, and catalytic capacities in the case of non-coding RNA (ncRNA) or coding RNA. Several studies on mRNA in eukaryotic cells have shown that RBPs, together with ncRNAs, direct and regulate the post-transcriptional fate of mRNA in the nucleus and cytoplasm in a dynamic and cell type-specific manner [117,118,119].

### Splicing

Alternative splicing of pre-mRNA is a crucial mechanism for enhancing protein diversity in humans, enabling the expression of proteins in a tissue-specific manner. This process generates multiple mature mRNAs with distinct structures and functions by selectively combining exons and eliminating (sometimes retaining) introns. Seven basic types of alternative splicing have been identified: exon skipping, alternative 5′-splice site, alternative 3′-splice site, mutually exclusive exons, intron retention, use of alternative promoters, and alternative polyadenylation [120]. The diversity of splicing events is influenced by the “strength” of splice sites, the concentration and combination of splicing factors, chromatin modifications, and RNA secondary structures. The core spliceosome, recruited by *cis*-acting elements and *trans*-acting factors, orchestrates both constitutive and alternative splicing. As previously mentioned, it is composed of five snRNAs (U1, U2, U4, U5, and U6), and numerous associated proteins that together form small nuclear ribonucleoproteins (snRNPs) [120]. These snRNPs facilitate the precise rearrangement of exons (and occasionally introns) by catalyzing two sequential transesterification reactions. In the first step, the 5′ splice site of the intron is cleaved to form a lariat structure through a linkage with the branch point adenosine. In the second step, the 3′ splice site is cleaved, resulting in the release of the intron lariat and the ligation of the adjacent exons [121].

Upregulation, downregulation, or mutations in splicing factors can disrupt the splicing process, leading to the premature termination of mRNA maturation, thereby affecting protein translation and protein levels, which may result in the development of diseases. Dysregulation of alternative splicing can lead to the production of tumor-associated isoforms that influence cellular activities including proliferation, cell death prevention, metabolism rewiring, angiogenesis promotion, invasion, metastasis, and drug resistance [122]. Splicing factors directly interact with specific oncogenic miRNAs to either facilitate or inhibit their expression. Conversely, miRNAs can modulate the expression of splicing factors and function as oncogenes [123].

## 4. MicroRNAs

### 4.1. Biogenesis of MicroRNAs

miRNAs have become increasingly relevant in medicine because of their key role in regulating gene expression and their involvement in various diseases, including cancer [124,125,126], cardiovascular disorders [127,128,129], and neurological conditions [130,131,132,133], as they modulate the stability and translation of target mRNAs [134].

In eukaryotic organisms, RNA Pol II is responsible for transcribing both protein-coding mRNAs and various small non-coding RNAs (sRNAs) from independent transcriptional units. In human cells, a significant proportion of sRNAs, estimated to be around one quarter of all of them, are derived from intronic regions and are also transcribed by Pol II [135]. These transcripts are several kilobases in length, contain uridine residues, feature a 5′-cap, are differentially expressed during development, and have been used to create fusion genes with reporter plasmids [136,137,138]. The remaining sRNAs are transcribed by RNA Pol III and organized into multicistronic transcripts in the human genome. These RNAs share the same genomic orientation but are not likely transcribed from the same promoter [139]. Small RNA transcripts are processed into two main functional species, mature miRNAs and siRNAs, which are 21–25 nucleotides long. miRNAs regulate developmental processes in various eukaryotic organisms by providing sequence-specific complementation in the 3-′untranslated region (3′-UTR) of mRNA, leading to translation repression and preventing progression to the next stage of germinal development. siRNAs, on the other hand, recruit ribonuclease complexes to cleave their specific target mRNAs [135,136,140,141].

The biogenesis of miRNAs begins within the cell nucleus. Primary miRNAs (pri-miRNA) are processed to generate an intermediate RNA called the miRNA precursor (pre-miRNA). The pre-miRNA is 60–110 nucleotides long and forms a secondary stem-loop-type structure. Pre-miRNA processing is mediated by the RNase III endonuclease Drosha, which cleaves the RNA near the base of the stem-loop structure [142,143,144,145,146,147,148]. Drosha operates as part of two distinct multiprotein complexes. The major complex includes a variety of RNA-associated proteins, such as RNA helicases, double-stranded RNA (dsRNA)-binding proteins, and nuclear heterogeneous ribonucleoproteins (hnRNPs), which may contribute to broader RNA processing functions. The core functional unit, known as the microprocessor complex, primarily consists of the RNase III enzyme Drosha and its essential cofactor DGCR8 (DiGeorge syndrome critical region 8), a dsRNA-binding protein that recognizes and stabilizes pri-miRNAs, enabling their precise cleavage [145,146]. In vivo and in vitro knockdown reconstitution studies have shown that components of the minor Drosha complex are necessary and sufficient to mediate mature miRNA from pre-miRNA transcripts [145,146,148]. The pre-miRNAs are subsequently exported to the cytoplasm by the Exportin-5 receptor in a process that requires the Ran-GTP (Ran guanine nucleotide exchanger) complex [149,150,151,152].

Once in the cytoplasm, pre-miRNAs are processed by the RNase III enzyme Dicer, which cleaves the stem-loop structure to produce a double-stranded RNA (dsRNA) approximately 21–25 nucleotides in length. This duplex is then incorporated into RISC and unwound into two single strands, one of which selectively functions as the mature guide strand [153,154,155,156]. Within RISC, the mature guide strand of the siRNA or microRNA directs the complex to complementary sequences in target mRNAs. Binding to a perfectly complementary target typically induces endonucleolytic cleavage of the mRNA, whereas partial complementarity, often seen in miRNA interactions, results in translational repression or mRNA destabilization [157,158].

Multiple studies have identified Dicer and Ago2 proteins as key components of RISC, shedding light on their roles in complex assembly and gene-silencing mechanisms [159,160,161,162,163,164]. Cloning of the human *Dicer* gene has enabled detailed characterization of its RNA-binding properties and RNase III enzymatic activity. Structural analyses have identified several functional domains of the Dicer protein, including two helicase domains, a protein-interacting domain (PID), a PAZ domain (involved in binding the 3′ end of small RNAs), two RNase III domains arranged in tandem, and a double-stranded RNA-binding domain (dsRBD) [165,166,167]. Ago2 is recognized as the only catalytically active member of the human Argonaute family, with its endonucleolytic activity residing in the PIWI domain, a structural and functional analog of RNase H [168,169,170,171]. Cloning and expression of *Ago2* cDNA have enabled the production of recombinant Ago2 protein, which has been widely used in in vitro assays to study its siRNA-guided cleavage activity on target mRNAs [172]. Although the minimal Ago2–siRNA complex can mediate target recognition and cleavage, several studies have demonstrated that it is not sufficient for full RISC functionality [159,173]. This suggests that additional protein cofactors are required for optimal gene-silencing activity. Furthermore, direct physical interactions between Dicer and Ago2 have been observed both in vitro and in vivo, reinforcing the notion that Dicer is not only involved in miRNA and siRNA processing but also plays a critical role in RISC assembly and function [174,175,176,177,178,179]. The discovery of the R2D2 protein in *Drosophila melanogaster* (fruit flies), RDE-4 protein in *C. elegans*, and TRBP protein in humans provided further insight into the molecular composition of RISC and the assembly process during the initiation phase of RNA transcript processing [174,179,180,181,182]. Additionally, flies with mutations in *dicer2* and *ago2* exhibited increased transposon expression, suggesting that an endogenous interference RNA pathway plays a role in transposon silencing, as previously observed in *C. elegans* [183,184]. In this context, it has been demonstrated that endogenous small interfering RNAs (endo-siRNAs) are short RNA molecules, typically 21 nucleotides in length, that play a pivotal role in gene regulation and defense mechanisms within the somatic cells of *Drosophila melanogaster*. These siRNAs are primarily derived from transposons and heterochromatic regions and are complementary to mRNAs. Their primary function is to silence detrimental genetic elements such as transposons, thereby ensuring the preservation of genomic integrity [185]. Endo-siRNA generation from structured loci has also been observed, with these transcripts folding into secondary stem-loop structures [186,187,188]. Overall, it has been shown that the accumulation of these endo-siRNAs depends on Dicer activity and Loquacious (Loqs) protein, which associates with Dicer and plays a role in endo-siRNA biogenesis, specifically in generating repeated, inverted, and complementary transcripts [189,190].

In mammals, the first reported endo-siRNA was detected in the nuclear-interspaced region of the L1 retrotransposon in cultured human cells [191]. L1 contains sense and antisense promoters at its 5′-UTR, which can drive bidirectional transcription, producing overlapping complementary transcripts that Dicer processes into siRNAs. Additionally, endo-siRNAs have been identified in mouse oocytes, and are derived from various genomic sources [192,193]. A subset of these endo-siRNAs has been found near protein-coding genes, which can pair with pseudogenes to form repeated inverted structures. Furthermore, some pseudogene sequences can generate antisense transcripts that pair with coding genes to produce endo-siRNAs [194]. These findings highlight how endo-siRNAs are complementary to coding mRNA and provide insights into the regulatory mechanisms of cellular gene expression mediated by endo-siRNAs.

### 4.2. MicroRNAs as Therapeutic Agents

Given their critical role in gene regulation, miRNAs have emerged as potent therapeutic agents. They can be harnessed to target specific mRNAs involved in diseases, such as cancer, cardiovascular disorders, and viral infections. Researchers can modulate gene expression with high specificity by mimicking or inhibiting specific miRNAs, thereby offering the potential for more precise and targeted treatments. The growing understanding of miRNA biogenesis and its involvement in cellular pathways highlights the potential of miRNAs as versatile tools for gene therapy and precision medicine, with a particular focus on cancer treatment [195]. For instance, the use of small interfering RNA in HPV-positive human cervical cancer cells targeting HPV16 E6 and E7 genes resulted in cell death through apoptosis and autophagy [196]. In addition, miRNAs have been used to treat conditions, such as Mixed Hyperlipidemia [197]. The applications of miRNAs in medicine are extensive, ranging from their use as diagnostic and prognostic biomarkers to their role as therapeutic agents [198].

## 5. mRNA Vaccines

Vaccines are critical for preventing infections and diseases by priming the immune system to recognize and combat foreign pathogens, including bacteria, viruses, and other microorganisms. According to the U.S. National Cancer Institute, vaccines enhance the natural ability of the immune system to protect the body against infections and mitigate the risks associated with certain types of damaged or abnormal cells such as cancer cells. Certain vaccines introduce a nonpathogenic protein fragment from a specific pathogen, whereas others introduce attenuated or dead bacteria or viruses, thereby stimulating an immune response. Scientists have designed a new type of vaccine based on mRNA [199]. mRNA vaccines deliver mRNA to cells without entering the cell nucleus or integrating into the DNA, ensuring that no alterations to the genomic DNA occur. These vaccines introduce a small segment of mRNA that encodes a viral protein, typically a portion of the protein found on the outer membrane of the virus [200]. This mRNA instructs cells to produce viral proteins that the immune system then identifies as foreign. In response, the immune system generates antibodies that help protect the body by recognizing and neutralizing specific viruses or pathogens as well as marking them for elimination. Once formed, these antibodies remain in the body even after the pathogen is cleared, enabling a swift immune response if re-exposure occurs. If a person encounters the virus after receiving the vaccine, antibodies can promptly recognize, bind, and neutralize the virus, thereby preventing severe illness.

The development of mRNA-based technologies represents a significant advancement in medicine, with innovations in delivery methods enabling the use of mRNA-based vaccines in a new treatment era. The rapid, potent, and transient nature of mRNA-encoded proteins makes mRNA vaccines highly promising for the treatment of various diseases, including infectious diseases, cancer, and monogenic disorders. For instance, the speed and scalability of mRNA-based therapeutics have been pivotal for the global response to the COVID-19 pandemic [201]. Additionally, mRNA vaccines have shown promise in the treatment of pancreatic cancer [202]. The incorporation of mRNA vaccines into cancer immunotherapy represents a promising strategy to address the complexities of the immune tumor microenvironment. The combination of advancements such as immune checkpoint inhibitors with cancer vaccines has demonstrated considerable potential [203].

Future progress is expected to enhance nanoparticle design using artificial intelligence, leading to the creation of biocompatible multifunctional nanoparticles. These innovations hold promise for more effective, personalized, and long-lasting cancer treatment, potentially revolutionizing the field in the near future. However, despite the promising potential of mRNA therapeutics, several challenges persist, including issues related to mRNA stability, the duration of protein expression, delivery efficiency, and target specificity. Hopefully, these challenges will soon be addressed with the advent of a new era in mRNA vaccine development, driven by research on chemical modifications in RNA, also known as epitranscriptional marks.

## 6. RNA Modifications (Epitranscriptional Modifications)

The chemical modifications of RNA, which are known as epitranscriptional modifications (derived from the Greek prefix “epi”, meaning “over” or “above”), were first identified many years ago. Nevertheless, it has only been in recent years that we have begun to understand their role in cells. These modifications include the deposition of chemical groups (methyl, acetyl, thiol, hydroxy groups, among others), isomerization, or structural modification of the RNA nucleosides adenosine (A), cytidine (C), uridine (U), and guanosine (G). The most common and well-studied epitranscriptional modifications are N6-methyladenosine (m6A), which involves the addition of a methyl group to the sixth nitrogen of adenosine; N5-methylcytosine (m5C), which involves the addition of a methyl group to the fifth carbon of cytidine; and pseudouridine (ψ), an isomer of uridine in which the uracil is attached via a carbon–carbon glycosidic bond instead of a nitrogen–carbon bond [204]. Other epitranscriptional marks include inosine (I), 7-methylguanosine (m7G), N1-methyladenosine (m1A), 3-methylcytidine (m3C), 4-acetylcytidine (ac4C), ribose methylation (2′-O-Me or Nm), 1-methylguanosine (m1G), 2′-O-methyladenosine (Am), and N6, N6-dimethyladenosine (m6,6A). Although the biological functions of most RNA modifications remain largely unknown, several recent studies have shown that epitranscriptional modifications regulate a wide range of RNA-mediated processes, including RNA biogenesis, splicing, polyadenylation, transportation, localization, stability, and translation in eukaryotes, bacteria, and archaea [205]. These modifications play a pivotal role in a wide range of biological functions, such as innate immune response, sex determination, stem cell differentiation, circadian clock, meiosis, stress response, and cancer [205,206,207,208,209,210].

Pseudouridine, the fifth nucleotide, was the first RNA chemical modification to be identified in yeast using paper and ion-exchange chromatography in 1957 [211]. Since then, over 300 naturally occurring ribonucleosides and ribonucleotides, including doubly modified residues, have been identified using high-resolution cryo-electron microscopy (cryo-EM) and liquid chromatography–mass spectrometry (LC-MS/MS) (https://genesilico.pl/modomics/modifications, accessed on 14 May 2025) [212]. Advances in sequencing technologies have enabled the development of methods to identify chemical modifications in mRNA, rRNA, tRNA, non-coding RNAs (ncRNAs), lncRNAs, snRNAs, snoRNAs, and pri-miRNAs [205,213]. Strategies such as photoactivatable ribonucleoside-enhanced crosslinking immunoprecipitation (PAR-CLIP), crosslinking and immunoprecipitation (miCLIP), enhanced UV crosslinking followed by immunoprecipitation (eCLIP), and methylated RNA immunoprecipitation (MeRIP) have been coupled with next-generation sequencing approaches (Modification-seq, PA-modification-seq, eCLIP-seq, MeRIP-seq, etc.) to map epitranscriptional modifications [214]. Base-specific chemical labeling was used, as was the case for pseudouridine (ψ) mapping. This approach is based on chemical labeling of ψ with N-cyclohexyl-N0-(2-morpholinoethyl) carbodiimide metho-p-toluenesulfonate (CMC). The resulting CMC-ψ adducts prevent reverse transcriptase (RT) from functioning and halt cDNA synthesis by one nucleotide before the ψ modification. Thus, ψ modifications can be identified via sequencing [111]. Recently, nanopore RNA direct sequencing and specialized analysis methods have enabled mapping of epitranscriptional marks on native RNA [215].

### 6.1. Writers

RNA-modifying proteins (RMPs), responsible for adding epitranscriptional modifications to RNA, are known as “writers”. The first identified writer enzyme was N6-methyladenosine (m6A) writer methyltransferase-like 3 (METTL3), which forms a complex with methyltransferase-like 14 (METTL14), Wilms’ tumor 1-associating protein (WTAP), and KIAA1429. Crystallographic analysis, in vitro methylation assays, mutagenesis, and knockdown, among other experimental strategies, have demonstrated that the METTL3/METTL4 complex is mainly responsible for m6A modifications [216,217,218]. m6A modifications have been identified in mRNA, miRNAs, lncRNAs, circular RNAs (circRNAs), and viral RNA [219,220].

The NOP2/Sun RNA methyltransferase (NSUN) family proteins and DNA methyltransferase-2 (DNMT2) are responsible for the addition of m5C modifications to rRNAs, tRNAs, mRNAs, mt-tRNAs, and viral RNAs [221]. NSUN2 and DNMT2 have site-specific functions that complement each other in catalyzing the addition of m5C to tRNA, as evidenced by double knockout experiments involving Nsun2 and Dnmt2. In these experiments, global loss of m5C was observed in double-knockout cells, but not in single-knockout murine cells. The loss of m5C in tRNA resulted in tRNA degradation and decreased protein synthesis [222]. NSUN2 was the first m5C writer to be identified and has been linked to m5C marks on tRNA, mRNA, rRNA, mitochondrial tRNA (mt-tRNA), lncRNA, and viral RNA [221]. NSUN2 has been identified as the predominant mRNA m5C writer [223].

Pseudouridine is present in all types of RNA, including rRNA, tRNA, snoRNA, snRNA, miRNA, lncRNA, and ncRNA [224]. This modification induces conformational changes that are crucial for RNA activity, mRNA stability, ribosome assembly, splicing, 3′ end processing, and protein synthesis [225]. The writer proteins responsible for catalyzing the isomerization of uridine to Ψ can be categorized into two groups: RNA-independent pseudouridine synthase (PUS) proteins and RNA-dependent synthase dyskerin (DKC). Human PUS proteins comprise a family of 12 proteins that have been identified based on their homology with yeast PUS proteins [86]. Several proteins, including PUS1, PUS7, RPUSD4, and TRUB1, have been shown to play major roles in catalyzing pseudouridine in mRNA [210,226,227]. Alternatively, the DKC protein deposits pseudouridine through base-pairing interactions between H/ACA snoRNAs (guide) and the target RNA [228].

It is noteworthy that although it was previously hypothesized that epitranscriptional marks are catalyzed co-transcriptionally, recent evidence has confirmed that m6A and Ψ are indeed co-transcriptionally added to mRNA [207,210,229,230].

### 6.2. Erasers

The enzymes responsible for the elimination of epitranscriptional marks from RNA are referred to as “erasers”. Recent research has indicated that m6A, m5C, and ac4C modifications are reversible [231,232,233], whether the same is true for other epitranscriptomic marks remains unsolved.

Fat mass- and obesity-associated protein (FTO) and alpha-ketoglutarate-dependent dioxygenase alkB homolog 5 (ALKBH5) are m6A erasers. Jia et al. (2011) [231] found that m6A is a primary substrate for FTO in nuclear RNA, catalyzing its demethylation. These studies demonstrated that a reduction in FTO levels through knockdown increased m6A levels in mRNA, whereas overexpression had the opposite effect. Similarly, the knockdown of ALKBH5 in HeLa cells increased the relative level of m6A in mRNA, whereas the opposite outcome was observed when ALKBH5 was overexpressed. A significant elevation in the m6A levels was detected in samples from Alkbh5-deficient mice, providing evidence that ALKBH5 is an RNA demethylase that functions on mRNA in mammals [232].

The protein that acts as an m5C eraser belongs to the TET family of enzymes, specifically, TET1, TET2, and TET3. These enzymes are Fe (II)- and α-ketoglutarate (α-KG)-dependent dioxygenases that catalyze the oxidation of RNA 5-methylcytidine (5mrC) to form oxidized analogs such as 5-hydroxymethylcytidine (5hmrC), 5-formylcytidine (5frC), and 5-carboxycytidine (5carC) [234,235,236].

To date, no eraser proteins of ψ have been identified. Nevertheless, it has been speculated that ψ may not be a reversible modification, as the C-C glycosidic bond in ψ appears to be inert.

### 6.3. Readers

A third type of epitranscriptional RMP is denoted as “readers”. These proteins play an important role in recognizing epitranscriptional modifications in RNA and in recruiting other effector proteins. The most studied reader proteins are those that bind m6A. Examples of m6A reader proteins include YT521-B homology (YTH)-domain family proteins (such as YTHDC1-2 and YTHDF1-3), ECT2, and IGF2BP family proteins, which are involved in a variety of functions, including mRNA stability, mRNA decay, recruitment of splicing factors, and 3′-UTR processing [237].

Aly/REF export factor (ALYREF) and YBX1 proteins have been identified as readers of m5C modifications. ALYREF is the main nuclear m5C reader that promotes mRNA export, splicing, and stabilization [223,238]. YBX1 also plays a role in mRNA stabilization [239]. Profilin-1 (PFN1) is currently the only protein identified as a pseudouridine reader [240]. Table 2 summarizes the writer, eraser, and reader proteins associated with m6A, m5C, and ψ modification.

### 6.4. Modified RNA and Immune Response

The innate immune system represents the initial line of defense against pathogens, including viruses. Viral ARN is typically detected by endosomal RNA sensors, such as Toll-like receptors (TLRs), or cytosolic RNA sensors, including RNA sensor retinoic acid–inducible gene I (RIG-I) and melanoma-differentiation-associated protein 5 (MDA-5). This activates the antiviral immune response, which is characterized by the production of pro-inflammatory cytokines and type I interferons (IFN) [245].

It has been demonstrated that in vitro transcribed RNA with m5C, m6A, Ψ, m5U, or s2U nucleoside modifications failed to elicit differential activation of TLR3, TLR7, and TLR8 compared to unmodified RNA, resulting in the abolishment of TNF-α, IL-8, and IFN-α production [246]. Moreover, other studies have shown that pseudouridine nucleoside-modified mRNAs exhibited a higher translation capacity than m6A-, m5C-, and m5U-modified mRNAs or unmodified mRNAs; consequently, the unmodified mRNAs were observed to elicit a more robust immune response than the pseudouridine-modified mRNAs [247]. Later studies demonstrated that incorporating pseudouridine into mRNA enhanced translation by diminishing PKR activation and phosphorylation of the transcription factor eIF-2α, a substrate of PKR [248].

In vitro, the presence of m6A and ψ nucleotides is associated with attenuated signaling, characterized by the reduced initiation of RIG-I-mediated immune responses [249]. Some authors have previously demonstrated that the depletion of METTL3 and METTL14 results in an increase in viral RNA recognition by RIG-I, thereby stimulating type I IFN production in viruses such as human metapneumovirus, HBV, and HCV [250,251]. Similarly, a single m6A modification of HIV-1-derived RNA oligos inhibited IRF3 and IRF7 activation and IFN I production in an RIG-I-dependent manner [252]. Interestingly, infection with SARS-CoV-2, HIV-1, and ZIKV has been observed to increase the global cellular content of m6A modifications [253,254,255]. Furthermore, it has been shown that these RNA viruses have acquired epitranscriptional modifications in their genomes [253,254,256,257,258,259,260,261,262,263,264,265,266]. Based on this principle, modified mRNA vaccines have been developed to overcome nuclease degradation and innate immunogenicity, thereby effectively expressing viral proteins and eliciting pathogen-specific cellular immune responses.

### 6.5. Modified mRNA Vaccines

The groundbreaking contributions of Katalin Karikó and Drew Weissman on pseudouridine and the replacement of uridine with N1-methyl-pseudouridine in RNA have significantly accelerated the development of mRNA-based vaccines, particularly the SARS-CoV-2 vaccine [267]. Notably, incorporation of N1-methyl-pseudouridine into synthetic mRNA has exhibited superior performance in terms of translation capacity, high-fidelity incorporation, and reduced TLR3 activation compared to Ψ [268,269,270].

Pfizer-BioNTech and Moderna Therapeutics have developed highly effective COVID-19 mRNA vaccines with over 90% efficacy in preventing the symptoms of infection [181]. These lipid nanoparticle-encapsulated nucleoside-modified mRNA (mRNA-LNP) vaccines utilize N1-methyl-Ψ, which substitutes all uridines in the mRNA sequence, resulting in the production of a full-length SARS-CoV-2 spike protein with two specific mutations (K986P and V987P) [271]. The SARS-CoV-2 mRNA vaccine is taken up by muscle and infiltrated immune cells, including dendritic cells and macrophages, where the spike protein is expressed on the cell surface, thereby triggering cellular and humoral immune responses. Another study evaluated the immunogenicity and efficacy of the CV0501 mRNA-based COVID-19 vaccine targeting the Omicron variant in Wistar rats. The results demonstrated that CV0501 elicited strong neutralizing antibody (nAb) responses against the Omicron BA.1 variant as well as other SARS-CoV-2 variants, including BA.2 and BA.5. Additionally, the vaccine induced a significant increase in the number of IFN-γ-producing T cells, highlighting its potential to generate a robust immune response [272].

Several studies have investigated the potential of mRNA-LNP vaccines as a novel approach for the development of prophylactic vaccines against a range of significant infectious diseases including IAV, ZIKV, DENV, HIV-1, and HPV. A concise overview of selected studies is presented below; however, comprehensive reviews of mRNA vaccines are available elsewhere [273,274].

The immunogenicity and protective efficacy of the full-length HA-encoding modified mRNA vaccine were evaluated in mice, rabbits, and ferrets. The animals were immunized with the vaccine and their corresponding specific antibody responses were assessed. A single immunization with the vaccine resulted in protection against homologous A/California/07/2009 and heterologous A/Puerto Rico/8/1934 viral challenges in mice. Furthermore, two immunizations with the vaccine induced protection against H5N1 influenza virus infection [275].

Research groups have endeavored to develop vaccines that protect against multiple pathogens. The AR-CoV/IAV mRNA vaccine was developed by combining two mRNA vaccines: ARCoV, which encodes the receptor-binding domain (RBD) of SARS-CoV-2, and ARIAV, which encodes the HA antigen of the influenza A virus (H1N1) [276]. This vaccine was administered to mice and its immunogenicity and protective efficacy were evaluated. The results demonstrated that AR-CoV/IAV immunization elicited an antigen-specific immune response characterized by Th1 cytokine-secreting CD4+ T cells and IFN-γ+ or TNF-α+ CD8+ T cells, which provided protection against influenza A virus and SARS-CoV-2 infection [276].

Another study evaluated the efficacy of the nucleoside-modified ZIKV mRNA-LNP vaccine, demonstrating that a single low-dose immunization with ZIKV pre-membrane and envelope (prM-E) mRNA-LNPs was protective in both mice and rhesus macaques, eliciting higher neutralizing antibody responses than a single immunization with purified inactivated virus (PIV) and plasmid DNA vaccines [277].

The immunogenicity and antitumor efficacy of mRNA-LNP cancer vaccines encoding a chimeric protein derived from the fusion of HPV-16 E7 oncoprotein and herpes simplex virus type 1 glycoprotein D (gDE7) were evaluated in mouse models of HPV-16–associated tumors [278]. The findings showed that a single low-dose immunization with any of the three mRNA-LNP formulations tested (U self-amplifying mRNA and U- or m1-pseudouridine non-replicating mRNA) induced high frequencies of IFN-γ+ CD8+ T cells in peripheral blood mononuclear cells (PBMCs), eradicated subcutaneous tumors at different growth stages, and generated memory T cell responses capable of preventing tumor relapse [278].

In a study conducted by Wollner et al. (2021), the safety and efficacy of a modified mRNA vaccine encoding membrane and envelope structural proteins from DENV serotype 1 encapsulated in lipid nanoparticles were assessed [279]. Interestingly, the vaccine encoding the prM and E proteins was found to trigger significant production of neutralizing antibodies and activate antiviral CD4+ and CD8+ T cells, resulting in potent antiviral immune responses in murine models. Furthermore, the study demonstrated that immunocompromised AG129 mice that received the vaccine exhibited protection when challenged with a lethal dose of DENV.

Significant advancements have been made in the understanding of the biology of HIV-1 infections. Despite these advances, effective vaccines have not yet been clinically developed. Therefore, a study was conducted to characterize the immune responses induced by an mRNA-LNP vaccine encoding clade C transmitted/founder HIV-1 envelope (Env) 1086C. Intradermal vaccination of rabbits and rhesus macaques with HIV-1 Env mRNA-LNP resulted in elicitation of high levels of gp120-specific antibodies. However, the administration of five vaccine injections did not yield the desired outcome of a broad and durable neutralizing antibody response, exemplifying the significant challenge posed by the development of effective HIV-1 vaccines [280].

## 7. Conclusions

**Figure 2:**
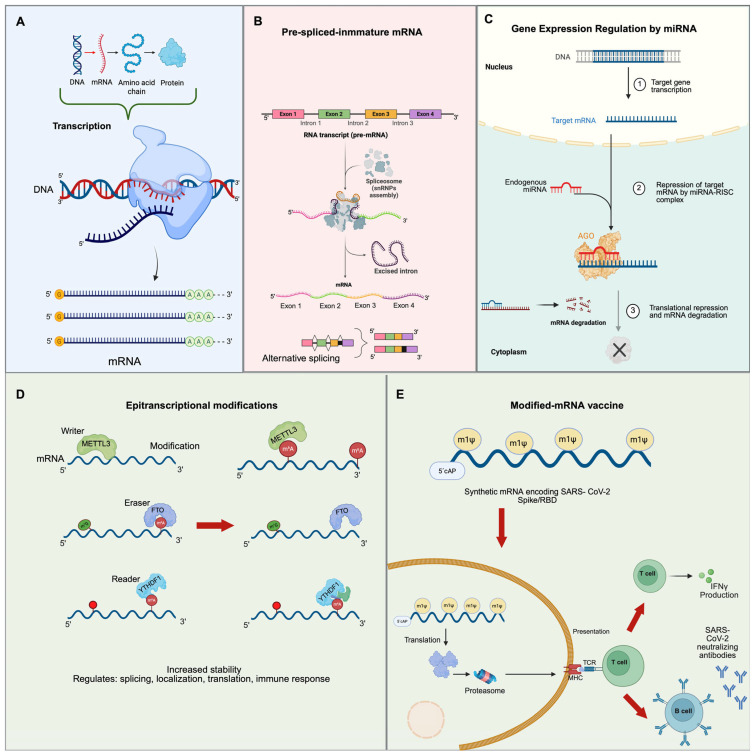
The mechanisms of RNA-mediated regulation of gene expression. (**A**) Transcription. The synthesis of RNA from a DNA template is catalyzed by RNA polymerase II. Transcription begins at the promoter region and proceeds along the DNA template strand, producing a precursor mRNA (pre-mRNA) that contains both exons and introns. (**B**) Splicing mechanism. During mRNA processing, non-coding intronic regions are typically removed, and coding exons are joined together to form mature mRNA. This process is mediated by the spliceosome, a large RNA–protein complex. In certain cases, introns are retained in the final transcript, a phenomenon known as intron retention, which can modulate gene expression and protein diversity. (**C**) Gene expression regulation by miRNAs. Double-stranded RNA (dsRNA) is processed by the endonuclease Dicer, which cleaves it into short interfering RNA (siRNA) duplexes. One strand of the duplex, known as the guide strand, is incorporated into the RNA-induced silencing complex (RISC). Within this complex, the AGO2 protein plays a central role in recognizing and binding to complementary sequences on target mRNAs. This leads to the cleavage and degradation of the target mRNA, resulting in post-transcriptional gene silencing. (**D**) Epitranscriptional modifications. Chemical modifications to RNA after transcription, are key regulators of RNA stability, splicing, translation, and degradation. Common modifications include N6-methyladenosine (m^6^A), 5-methylcytosine (m^5^C), N1-methyladenosine (m^1^A), 7-methylguanosine (m^7^G), and pseudouridine. Among these, m^6^A is the most extensively studied. It is catalyzed by a “writer” complex composed of METTL3, METTL14, and WTAP. This modification can be dynamically removed by “eraser” enzymes such as FTO. Recognition of m6A marks by “reader” proteins like YTHDF1 enables recruitment of additional effector proteins that modulate mRNA metabolism and gene expression. (**E**) Modified mRNA vaccine: Synthetic mRNA vaccines, such as those developed by Pfizer-BioNTech and Moderna, encode the receptor-binding domain (RBD) of the SARS-CoV-2 spike (S) protein. Upon cellular uptake, the mRNA is translated into protein and presented to T cells, initiating an immune response. The incorporation of N1-methylpseudouridine (m^1^Ψ) into the mRNA enhances its stability, reduces innate immune detection, and promotes efficient translation, resulting in stronger and more durable immune responses, including the production of neutralizing antibodies.

RNA is essential for the emergence of life, likely functioning as both a catalyst and genetic information store in primitive life forms. Moreover, RNA can regulate its expression through various mechanisms (Figure 2).

For instance, miRNAs fine-tune gene expression (Figure 2A), whereas splicing is essential for generating diverse protein isoforms from a single gene (Figure 2B). Both processes influence various cellular activities including development and disease progression. Small interfering RNA (siRNA) silencing of gene expression (Figure 2C) has been used to treat several diseases, including cancer [281]. Furthermore, RNA modifications, which influence RNA stability and translation, play a significant role in regulating gene expression and maintaining cellular homeostasis (Figure 2D). Recently, the understanding of these functions has led to groundbreaking technologies, such as modified mRNA vaccines, which utilize RNA’s ability to direct cells to produce protective proteins, offering an innovative method for fighting infectious diseases (Figure 2E).

In conclusion, RNA is not merely a passive intermediary between DNA and proteins; rather, it is a dynamic participant in the origin and maintenance of life. Its unique structural versatility enables it to fold into intricate three-dimensional conformations, allowing it to perform a wide range of biological functions. RNA’s capacity for self-replication, its central role in protein synthesis, and its ability to evolve and respond to environmental changes have positioned it at the core of many of the most significant advances in modern medicine. In recent years, RNA has gained prominence as a powerful tool in human therapeutics, contributing to molecular diagnostics, the discovery of prognostic biomarkers, and the development of novel vaccines and treatment strategies. Thus, RNA not only underpins fundamental biological processes but has also become indispensable in combating diseases.

## Figures and Tables

**Table 1:** Key Features of RNA.

Feature	Description
Genetic Information	RNA can store and transmit genetic information like DNA. Its sequence of nucleotides encodes information and can be replicated.
Catalytic Properties	Certain RNA molecules, known as ribozymes, can catalyze biochemical reactions. This suggests that RNA could facilitate critical metabolic processes even without proteins.
Self-Replication	Some RNA sequences can undergo self-replication under certain conditions, supporting the idea that RNA could have played a role in early life forms that required a means to reproduce.

**Table 2:** Writers, erasers, and readers of three most abundant epitranscriptional marks in RNA.

Modification	Known Functions	Writers	Erasers	Readers
m6A	Regulates: splicing, stability localization, and translation of mRNA [241]. Immune response [242]	-METTL3-METTL14-WTAP-complex	-FTO -ALKBH5	YTHDC1-2 YTHDF1-3
m5C	Regulates: stability of tRNA; ribosome biogenesis; stability, localization, and translation of mRNA [243]. Immune response [244]	-NSUN1-7 -DNMT2	-TET1-3, -ALKBH1	ALYREF, YBX1
Ψ	Regulates: stability of tRNA; ribosome biogenesis; translation of mRNA. Immune response [228]	-RNA-independent Pseudouridine synthases: PUS1, PUSL1, PUS3, TRUB1, TRUB2, PUS7, PUS7L PUS10, RPUSD1-4 -RNA-dependent Pseudouridine synthase: DKC1	?	PFN1
